# Identification of biomarkers related to copper metabolism in patients with pulmonary arterial hypertension

**DOI:** 10.1186/s12890-023-02326-6

**Published:** 2023-01-23

**Authors:** Lei Wang, Wei Zhang, Cong Li, Xin Chen, Jing Huang

**Affiliations:** 1 Department of Respiratory and Critical Care Medicine, The Second Affiliated Hospital of Xi’an Jiaotong University (Xibei Hospital), Xi’an, 710004 Shaanxi China; 2 Department of Emergency, The First Affiliated Hospital Xi’an Jiaotong University, Xi’an, 710061 Shaanxi China; 3 Department of Radiology, The Second Affiliated Hospital of Xi’an Jiaotong University (Xibei Hospital), Xi’an, 710004 Shaanxi China; 4 Department of Rheumatism and Immunology, The First Affiliated Hospital Xi’an Jiaotong University, Xi’an, 710061 Shaanxi China

**Keywords:** Pulmonary arterial hypertension, Copper metabolism-related genes, Biomarkers

## Abstract

**Background:**

The pathogenesis of pulmonary arterial hypertension (PAH) and associated biomarkers remain to be studied. Copper metabolism is an emerging metabolic research direction in many diseases, but its role in PAH is still unclear.

**Methods:**

PAH-related datasets were downloaded from the Gene Expression Omnibus database, and 2067 copper metabolism-related genes (CMGs) were obtained from the GeneCards database. Differential expression analysis and the Venn algorithm were used to acquire the differentially expressed CMGs (DE-CMGs). DE-CMGs were then used for the coexpression network construction to screen candidate key genes associated with PAH. Furthermore, the predictive performance of the model was verified by receiver operating characteristic (ROC) analysis, and genes with area under the curve (AUC) values greater than 0.8 were selected as diagnostic genes. Then support vector machine, least absolute shrinkage and selection operator regression, and Venn diagrams were applied to detect biomarkers. Moreover, gene set enrichment analysis was performed to explore the function of the biomarkers, and immune-related analyses were utilized to study the infiltration of immune cells. The drug-gene interaction database was used to predict potential therapeutic drugs for PAH using the biomarkers. Biomarkers expression in clinical samples was verified by real-time quantitative PCR*.*

**Results:**

Four biomarkers (DDIT3, NFKBIA, OSM, and PTGER4) were screened. The ROC analysis showed that the 4 biomarkers performed well (AUCs > 0.7). The high expression groups for the 4 biomarkers were enriched in protein activity-related pathways including protein export, spliceosome and proteasome. Furthermore, 8 immune cell types were significantly different between the two groups, including naive B cells, memory B cells, and resting memory CD4 T cells. Afterward, a gene-drug network was constructed. This network illustrated that STREPTOZOCIN, IBUPROFEN, and CELECOXIB were shared by the PTGER4 and DDIT3. Finally, the results of RT-qPCR in clinical samples further confirmed the results of the public database for the expression of NFKBIA and OSM.

**Conclusion:**

In conclusion, four biomarkers (DDIT3, NFKBIA, OSM, and PTGER4) with considerable diagnostic values were identified, and a gene-drug network was further constructed. The results of this study may have significant implications for the development of new diagnostic biomarkers and actionable targets to expand treatment options for PAH patients.

**Supplementary Information:**

The online version contains supplementary material available at 10.1186/s12890-023-02326-6.

## Introduction

Pulmonary hypertension (PH) is defined by a mean pulmonary arterial pressure ≥ 20 mmHg at rest as assessed by right heart catheterization. PH is a devastating vascular disease characterized by remodeling of pulmonary arteries, elevated pulmonary artery pressure, and subsequent development of right heart failure. Pulmonary arterial hypertension (PAH; World Health Organization Group 1) represents a specific subset of this disease that is focused on the lung vasculature, the most common types of PAH are idiopathic PAH and PAH associated with connective tissue disease [1]. Without effective treatment, PAH results in high morbidity and mortality [2], and early and accurate diagnosis of PAH is critical to patient prognosis. The development of comprehensive mechanistic theories for PAH may improve our understanding of the disease and facilitate the development and translation of effective therapies and biomarkers.

The gold standard approach to confirm PAH is right heart catheterization, but this is an invasive test that is not easily accepted by patients, so the development of biomarkers is an ongoing quest to improve outcomes. Researchers determined that none of the blood biomarkers identified in149 articles provide enough accuracy to replace current diagnostic approaches, either due to a lack of data or a lack of specificity [3]. To move closer towards precision medicine for PAH, there is an urgent need to identify novel biomarkers with high value. Increasing evidence has shown that a multiple-biomarker approach could be superior to using a single biomarker.

Recently, the metabolic theory of PAH has emerged, which facilitates the identification of several key metabolic targets that are directly involved in PAH pathogenesis and can form the basis of biomarker and drug discovery programs [4]. Metabolic changes occur in PAH pulmonary arteries, including abnormalities in glycolysis and glucose oxidation, fatty acid oxidation, glutaminolysis, arginine metabolism, one-carbon metabolism, and the tricarboxylic acid cycle; PAH-associated nuclear and mitochondrial mutations can also affect metabolism [5, 6]. Copper metabolism has also become an emerging metabolic research direction. Copper is one of the most abundant basic transition metals in the human body; both excess copper levels and copper deficiency can be harmful, and careful homeostatic control via copper metabolism is important [7]. In addition, recent exciting work has implicated copper-handling and copper-utilizing proteins in controlling the striking metabolic changes that occur in proliferating cells [8], indicating a potential role of copper metabolism in PAH pathogenesis. It was indeed proven that copper could be a biomarker for PAH [9]. Copper also plays a significant role in the control of endothelial cell proliferation in PAH [10], but the mechanisms and genes related to copper metabolism that are involved in PAH development are still not clear. Copper participates in PAH development, but the role of specific genes related to copper metabolism in pathogenesis of PAH remain to be determined, which would help to identify potential treatment targets and biomarkers.

Based on this rationale, in this study, PAH-related datasets were downloaded from the GEO database, and copper metabolism-related genes were obtained from the GeneCards database. Bioinformatics analysis methods such as limma, weighted gene coexpression network analysis (WGCNA), support vector machine (SVM), least absolute shrinkage and selection operator (LASSO) regression, and Venn diagrams were used to identify copper metabolism-related genes with diagnostic value for PAH. This study will increase our knowledge of the basic pathologic mechanisms behind vascular pulmonary disease, contribute to the early diagnosis and differentiation of PAH from other diseases and improve risk assessment before and during treatment using novel copper metabolism-related biomarkers. In the best-case scenario, the findings might even help in individualizing prevention and treatment. We present the following article in accordance with the TRIPOD reporting checklist.

## Materials and methods

### Data source

The PAH-related GSE33463 and GSE113439 datasets and the corresponding sample grouping information were downloaded from the GEO database (https://www.ncbi.nlm.nih.gov/geo/). Among them, the GSE33463 dataset with 71 samples (PAH: Control = 30: 41) was used as the training set, and the GSE113439 dataset with 17 samples (PAH: Control = 6: 11) was used for the validation of the diagnostic model. In addition, 2067 CMGs were obtained from the GeneCards database (https://www.genecards.org/) with "copper metabolism" as keywords.

### Identification of DE-CMGs and functional enrichment analysis

The "limma" package [11] was used to perform differential analysis to obtain DEGs based on 31 PAH samples and 40 control samples from the GSE33463 dataset (|log_2_(fold change)(FC)|> 0.5 and *p* value < 0.05). Furthermore, to screen the DE-CMGs, the "ggvenn" package was used to perform Venn analysis on the DEGs and CMGs. Subsequently, Gene Ontology (GO) annotation and Kyoto Encyclopedia of Genes and Genomes (KEGG) functional enrichment of DE-CMGs were analysed by the "clusterProfiler" package [12–15], and visualized by the "ggplot2" package.

### Identification of DE-CMG modules significantly associated with PAH using WGCNA

The DE-CMG expression matrices of 71 samples in GSE33463 were used as input data for WGCNA using the "WGCNA" package [16], and PAH and control were used as trait data to construct a coexpression network. First, all samples were clustered, and redundant samples were eliminated. Sample clusters and trait heatmaps were built, and the optimal soft threshold was determined. The modules were divided by the dynamic cutting tree algorithm, and the parameter minModuleSize was set to 10 to obtain the gene module. Correlation analysis was performed to determine the relationship between the modules and PAH and those modules with strong correlations were selected for subsequent analysis.

### Construction and validation of diagnostic models

Based on the modules with strong correlations obtained from WGCNA, genes in the module were identified as DE-CMGs. Additionally, the roc function of the "pROC" package [17] was used to draw ROC curves based on the grouping information of the sample and the expression level of each module DE-CMG to select candidate key genes. The 41 control samples and 30 PHA samples in the GSE33463 dataset were randomly divided into training and testing sets at a ratio of 6:4. In the training set, to examine the impact of different candidate key gene combinations on the diagnostic efficiency, the possible combinations of all candidate key genes were calculated by SVM. The combination with the highest predictive rate for diagnostis (i.e., indicating the genes in the combination are biomarkers) was selected, and a model was built in the training set. The model classification ability was verified using a tenfold cross-validation method, and the sensitivity, specificity, negative predictive value, and positive predictive value of the model for predicting pulmonary hypertension were analysed. LASSO regression was also utilized to screen the candidate biomarkers based on key genes, and tenfold cross-validation was adopted to verify the model. Biomarkers were further detected by intersecting the results of SVM and LASSO regression using a Venn diagram. In addition, the predictive performance of the model was verified by ROC curves and the sensitivity and specificity of the model in the testing set and GSE113439 dataset.

### GSEA of biomarkers

To explore the function of the diagnostic genes, the 30 PAH patient samples were divided into high and low expression groups according to the median expression of the diagnostic genes, and GSEA was performed on all genes. The FDR < 25% and NOM.*p* value < 0.05 were set as significace thresholds.

### Immune cell infiltration accessment using the CIBERSORT algorithm

To investigate the immune cells infiltration in the control group and PAH group, the Cell type Identification By Estimating Relative Subsets Of RNA Transcripts (CIBERSORT) algorithm and the LM22 gene set were used to calculate the proportions of 22 immune cell types in 71 samples (Control: PAH = 41:30). The proportion of each immune cell type in each sample was calculated using the CIBERSORT algorithm, and the samples with *p* > 0.05 were excluded (remaining samples control: PAH = 41: 30), according to the statistical value. According to the scores of each immune cell in the two groups, a score heatmap of 22 immune cell types was drawn. The "ggplot2" package was used to draw boxplots with the Wilcoxon rank sum test method. Then, a correlation heatmap of the biomarkers and 22 immune cell types was plotted, and the two immune cell types that were most positively or negatively correlated with the biomarkers were selected for further analysis of the differences in immune cell expression and their corresponding biomarkers between the control and PAH groups.

### Biomarker potential drug prediction using the DGIdb database

The DGIdb (https://dgidb.genome.wustl.edu/) database was used to predict the potential therapeutic drugs for PAH using the biomarkers, and Cytoscape software [18] was used to visualize the prediction results.

### Validation of biomarker expression

To further confirm the results of the public database analysis, we collected eight control peripheral blood mononuclear cell (PBMC) samples from healthy subjects and eight PBMC samples from patients with PAH (the basic characteristics of the patients are shown in Additional file 1: Table S1) and isolated RNA for RT-qPCR. Doppler echocardiogram was performed to screen for the presence of PAH. Pulmonary artery systolic pressure (sPAP) was estimated adopting a modified Bernoulli equation [19]: sPAP = 4 x (tricuspid systolic jet)^2^ + 10 mmHg (estimated right atrial pressure). PAH was defined as an estimated sPAH > 35 mmHg using echocardiograms. Total RNA was separated by TRIzol (Ambion, Austin, USA) based on the manufacturer’s guidance. The inverse transcription of total RNA into cDNA was implemented using the first strand cDNA synthesis kit (Servicebio, Wuhan, China) based on the manufacturer’s instructions. Then, qPCR was carried out utilizing 2 × Universal Blue SYBR Green qPCR Master Mix (Servicebio, Wuhan, China) according to the manufacturer’s instructions. The primer sequences for PCR are listed in Table 1. Expression levels were normalized to the internal reference GAPDH and computed employing the 2^−ΔΔCq^ formula.

**Table 1:** The primer sequences for qPCR

Primer	Sequence
DDIT3 For	TCACCACTCTTGACCCTGCTTC
DDIT3 Rev	TGACCACTCTGTTTCCGTTTCC
NFKBIA For	GAGGAGTACGAGCAGATGGTCAA
NFKBIA Rev	CAATTTCTGGCTGGTTGGTGAT
OSM For	CACAGACTGGCCGACTTAGAGC
OSM Rev	TGAGTGCATGAAGCGATGGTAG
PTGER4 For	CAGCAGTACATCTCAGACCCTCC
PTGER4 Rev	ACCAGCCTCATCCACCAGTAA
GAPDH For	CCCATCACCATCTTCCAGG
GAPDH Rev	CATCACGCCACAGTTTCCC

### Statistical analysis

All statistical analyses were performed using R software (version 4.0.3). The differences between the two groups were compared by the Wilcoxon test. P < 0.05 was considered statistically significant.

## Results

### Identification of DE-CMGs and functional enrichment analysis

**Fig. 1:**
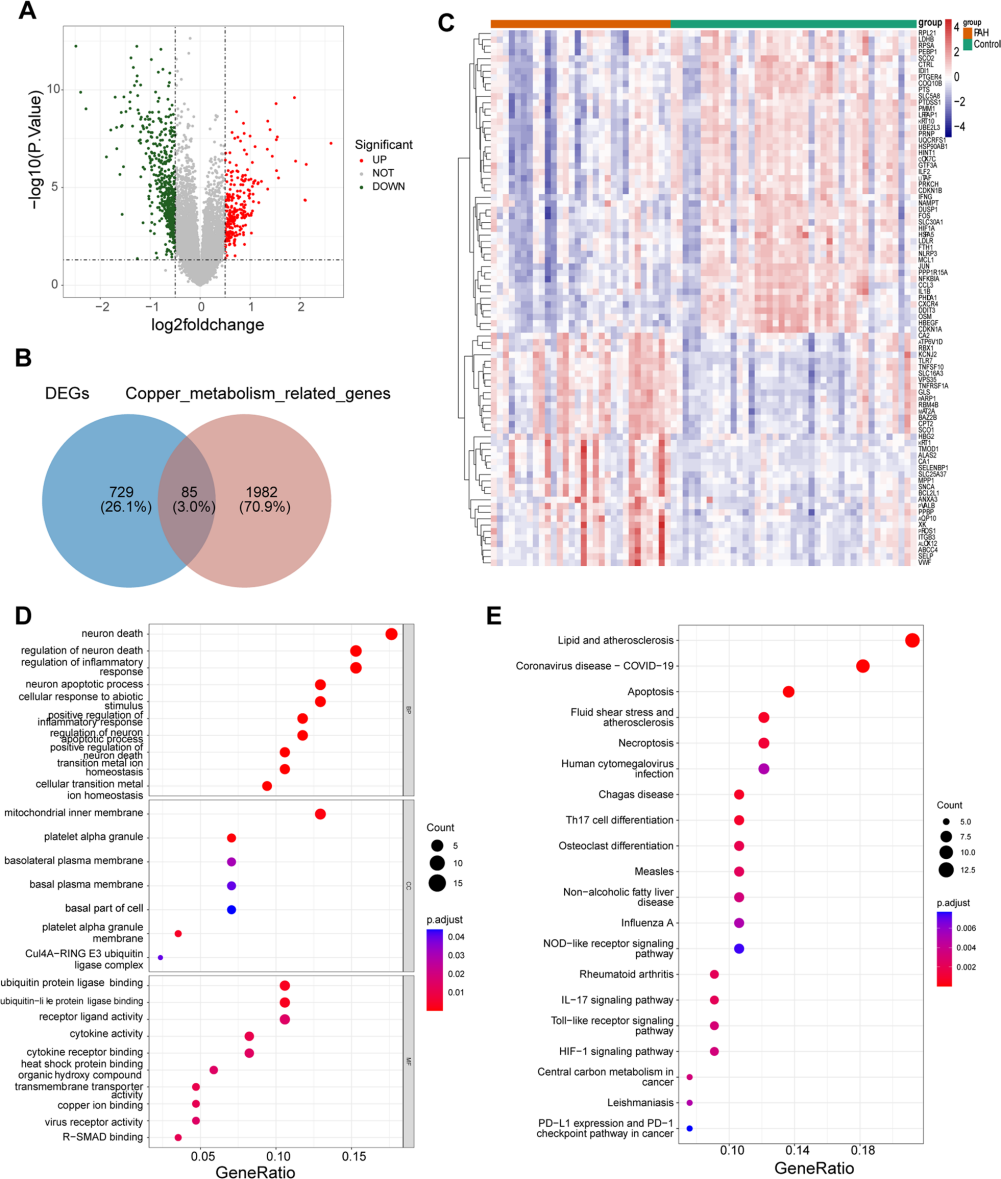
Identification of 85 DE-CMGs and their enrichment analysis. **A** 814 DEGs including 258 up-regulated (red dots) and 556 down-regulated (green dots) genes from the GSE33463 dataset in the volcano map. **B** Venn diagram to detect 85 DE-CMGs. **C** Heatmap of the expression of the top 100 DEGs. **D** The top 27 GO terms included 10 biological process (BP) terms, 7 cellular component (CC) terms, and 10 molecular function (MF) terms of the DE-CMGs. **E** Top 20 KEGG pathways of the DE-CMGs

A total of 814 DEGs were obtained from the PAH vs. control comparison group, including 258 up-regulated genes and 556 down-regulated genes (Fig. 1A). Moreover, 85 DE-CMGs were identified from the overlap analysis of DEGs and CMGs (Fig. 1B–C). These DE-CMGs were enriched in 445 biological process (BP) terms, 7 cellular component (CC) terms, 22 molecular function (MF) terms, and 55 KEGG signalling pathways. These DE-CMGs were primarily enriched in various inflammatory response, neuron death and apoptotic, ion binding and homeostasis, protein transport and binding GO terms, such as regulation of inflammatory response, regulation of neuron death, neuron apoptotic process, transition metal ion homeostasis and copper ion binding (Fig. 1D). The DE-CMGs were primarily enriched in various immune- and disease-related KEGG pathways, such as Th17 cell differentiation, the IL-17 signaling pathway, coronavirus disease-COVID-19, and Chagas disease (Fig. 1E).

### Identification of DE-CMG modules significantly associated with PAH using WGCNA

**Fig. 2:**
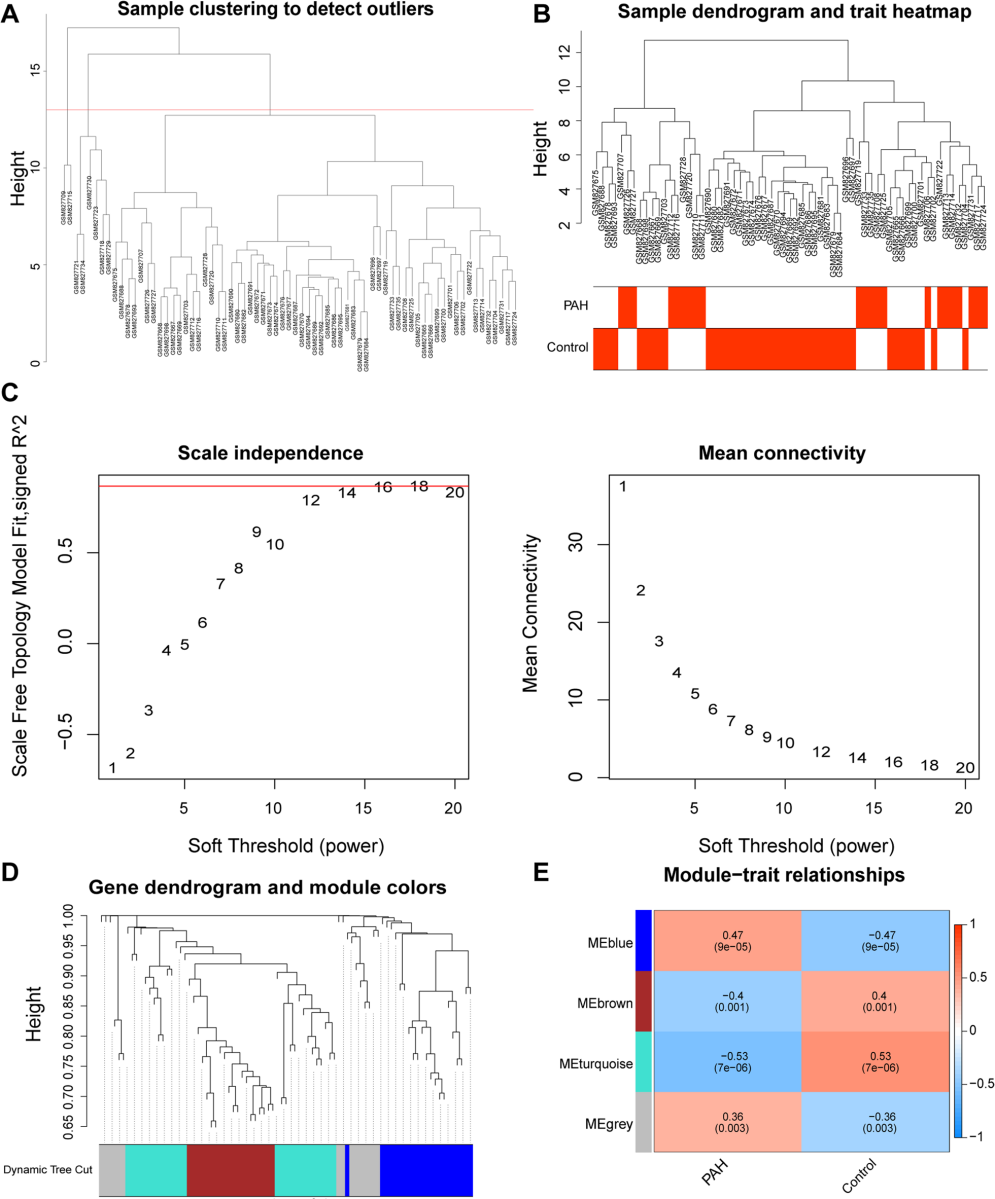
Construction of WGCNA to identify DE-CMG modules in the GSE33463 dataset. **A** Cluster dendrogram of module eigengenes to detect outlier samples. **B** Dendrogram of all expressed genes in the PAH and control samples clustered based on a dissimilarity measure (1‐TOM). **C** Analysis of the scale-free topology fit index and the mean connectivity for various soft-threshold powers (β) for the genes. **D** Hierarchical clustering tree based on the topological overlap dissimilarity (1-TOM). **E** Heatmap of the module-trait relationships. The corresponding P values are also annotated

To identify the DE-CMG modules significantly associated with PAH, the WGCNA was performed. Among the 71 samples of the GSE33463 dataset, 8 outlier samples (GSM827709, GSM827715, GSM827721, GSM827734, GSM827730, GSM827723, GSM827718 and GSM827729) were eliminated (Fig. 2A–B). The optimal soft threshold was determined to be 16 (R^2^ = 0.86) (Fig. 2C), and 4 gene modules were screened (Fig. 2D). Among the 4 gene modules, the turquoise module had a significantly strong correlation with PAH (Fig. 2E).

### Construction of a diagnostic model based on 4 genes

**Fig. 3:**
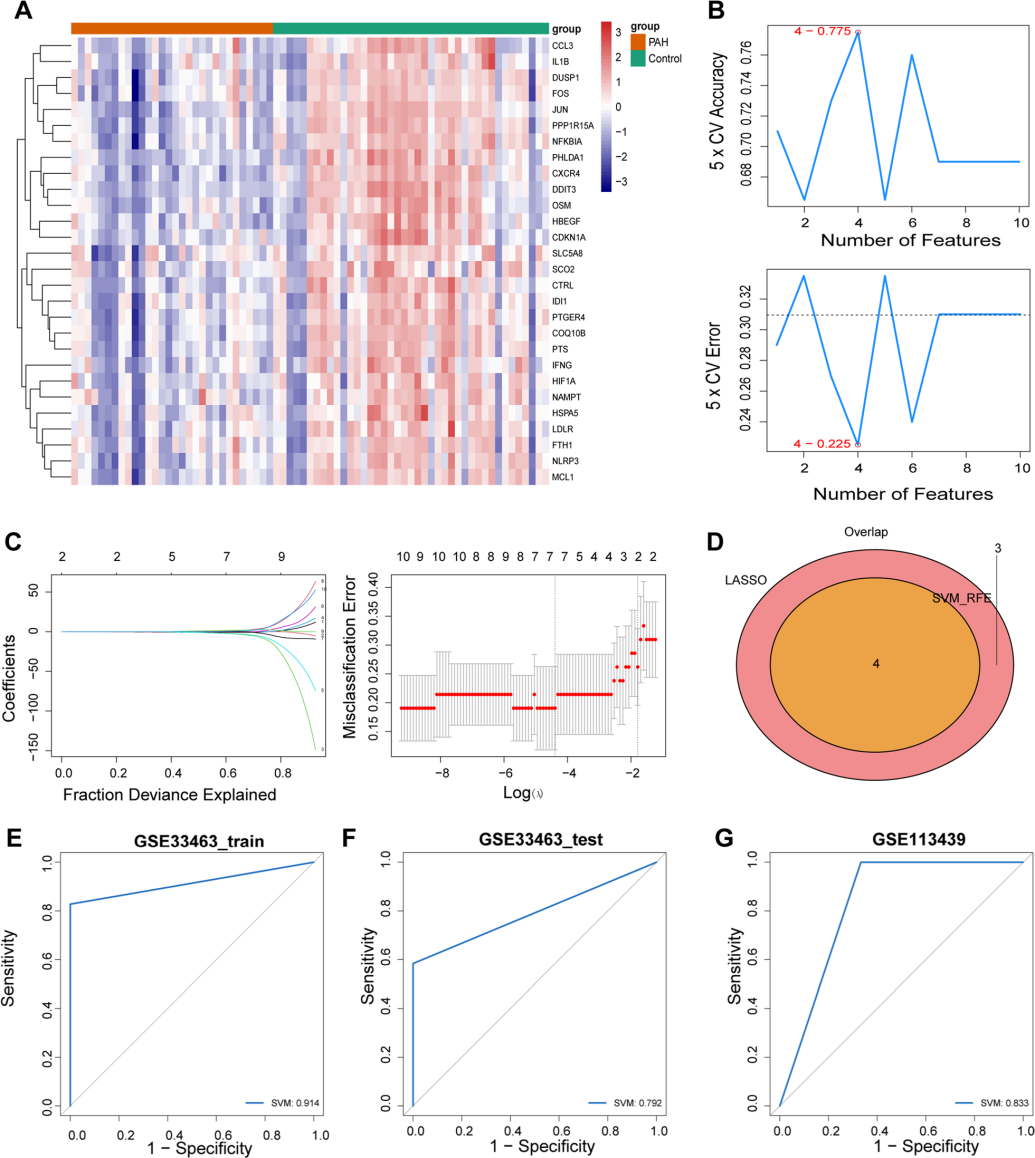
Identification and validation of four biomarkers. **A** Heatmap of the expression of 28 module DE-CMGs in PAH and control samples from the training set. **B** The accuracy and error of estimate generation for the SVM‐RFE algorithm in the training set. (C) Candidate genes selected by the LASSO regression model. **D** Four biomarkers detected by Venn diagram. ROC curves of the prognostic values of the four biomarkers in the training (**E**), testing (**F**), and GSE113439 (**G**) sets

**Fig. 4:**
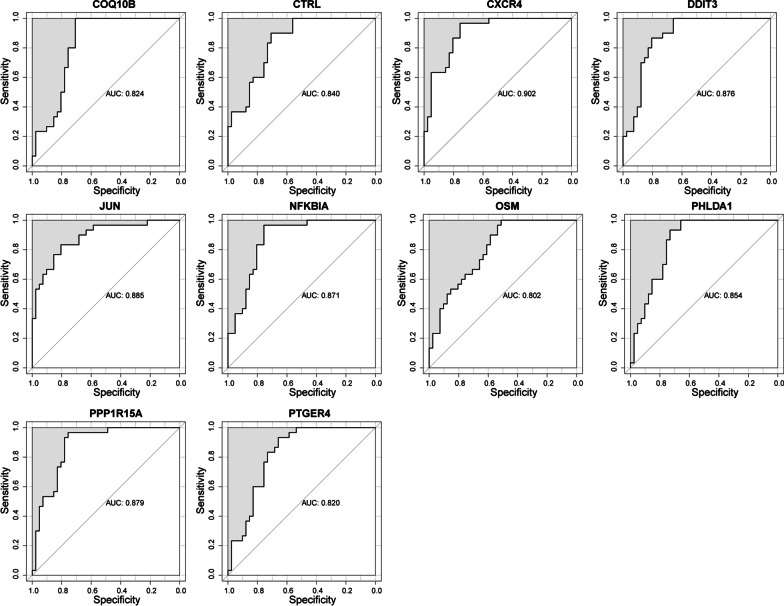
Ten module DE-CMGs with AUC values > 0.8

A total of 28 module DE-CMGs were acquired from the WGCNA, and the expression of these genes was significantly different in the PAH and control groups (Fig. 3A). Furthermore, 10 module DE-CMGs (CXCR4, JUN, DDIT3, PPP1R15A, NFKBIA, PHLDA1, CTRL, OSM, PTGER4, and COQ10B) were screened as candidate key genes according to AUC > 0.8, and the 95% confidence intervals of 28 module DE-CMGs are shown in Additional file 2: Table S2, indicating that these 10 candidate key genes had diagnostic value (Fig. 4). The combination of 4 genes (DDIT3, NFKBIA, OSM, and PTGER4) was filtered by SVM (Fig. 3B). Seven candidate genes (CXCR4, JUN, DDIT3, NFKBIA, OSM, PTGER4, and COQ10B) were filtered using LASSO regression (Fig. 3C). By intersecting the 4 genes filtered by SVM and the 7 genes detected by LASSO regression, 4 biomarkers (DDIT3, NFKBIA, OSM, and PTGER4) for the diagnosis of PAH were identified (Fig. 3D). The combination of the 4 genes distinguished PAH well from the control (Table 2), and the AUC value was greater than 0.7 in the training set, testing set, and GSE113439 dataset respectively, indicating high predictive effectiveness of the model (Fig. 3E–G).

**Table 2:** Construction and validation of the predictive performance of the four-gene model

Terms	Training dataset		Testing dataset		GSE113439 dataset	
	Actual disease	Actual normal	Actual disease	Actual normal	Actual disease	Actual normal
Predicted disease	13	5	17	5	4	0
Predicted normal	0	24	0	7	2	11
Total	13	29	17	12	6	11
Correct	13	24	17	7	4	11
Senstivity	100		100		67	
(%)						
Specificity		83		58		100
(%)						

### GSEA biomarker enrichment analysis

**Fig. 5:**
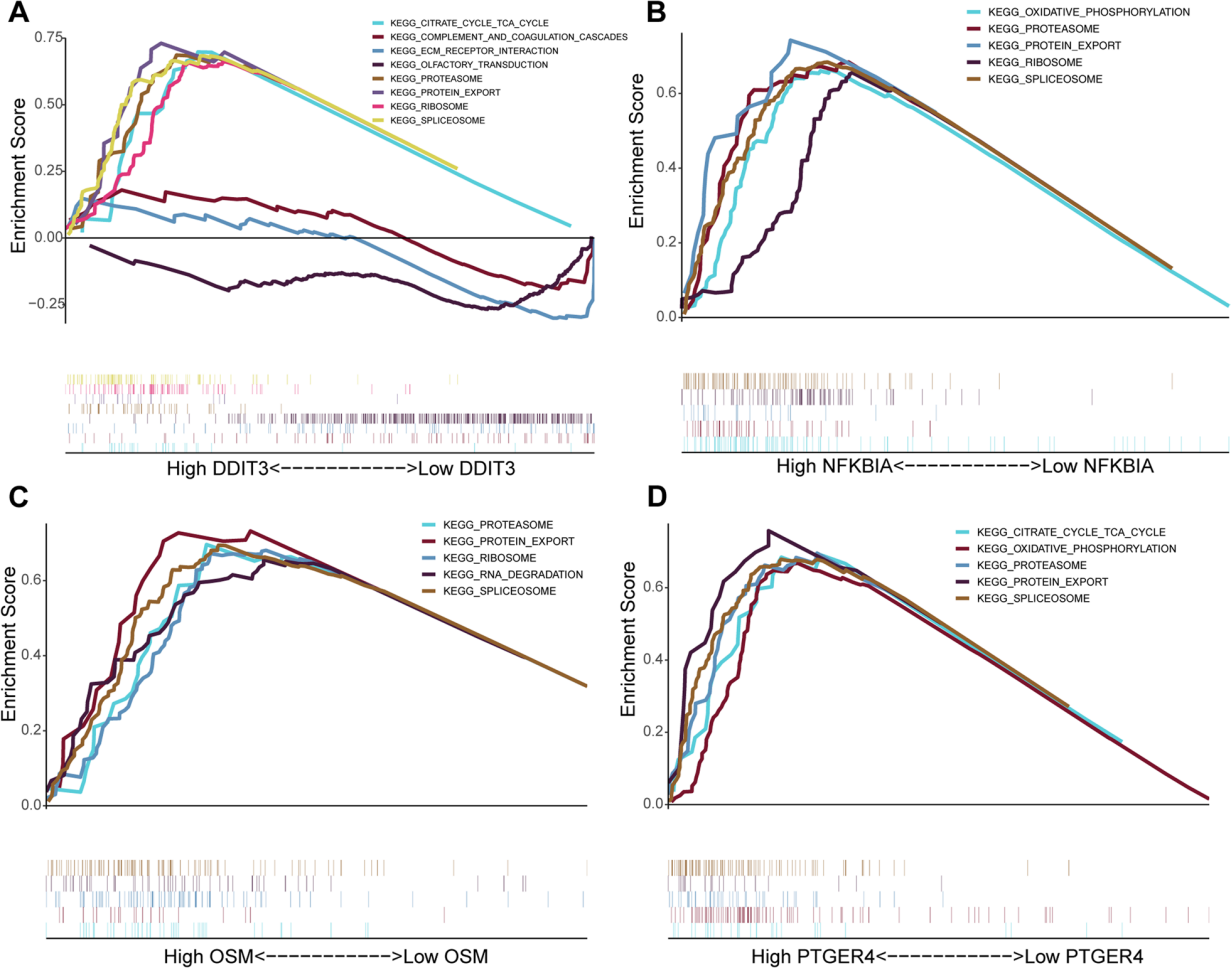
Top 5 KEGG pathways of the four biomarkers. Top 5 KEGG pathways of DDIT3 (**A**), NFKBIA (**B**), OSM (**C**), and PTGER4 (**D**) by GSEA enrichment

The top 5 KEGG pathways that were significantly enriched in the high and low expression groups for the 4 biomarkers are shown in Fig. 5. Protein export, spliceosome, citrate cycle TCA cycle, and proteasome were enriched in the high expression groups. In the low DDIT3 expression group, the genes were mainly enriched in the complement and coagulation cascades, ECM receptor interaction, and olfactory transduction pathways.

### Immune cell infiltration accessment by the CIBERSORT algorithm

**Fig. 6:**
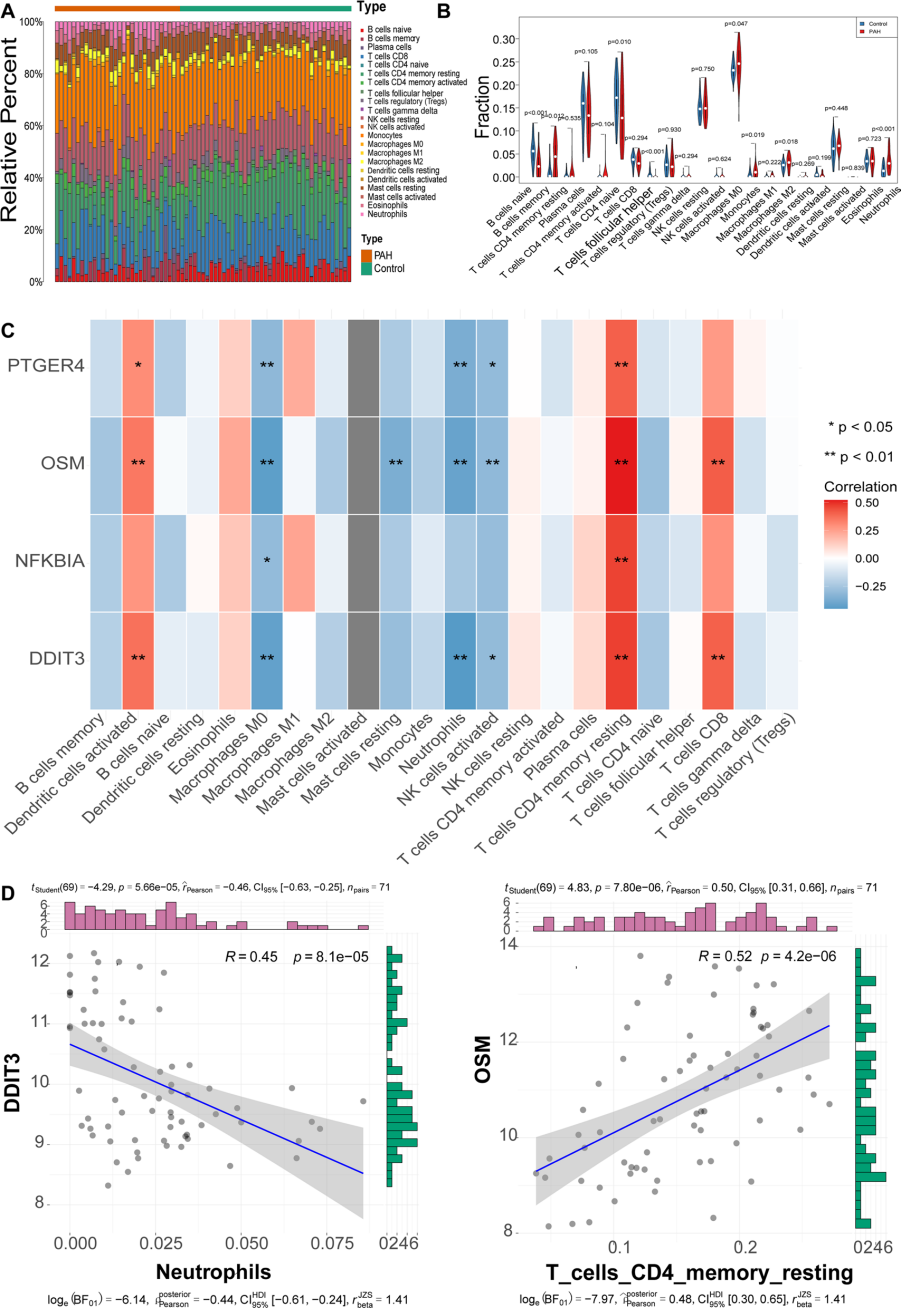
Evaluation of immune cell infiltration using the CIBERSORT algorithm. **A** A stacked bar plot of the proportions of 22 immune cell types in control and PAH samples from the training based on the CIBERSORT algorithm. **B** Vioplot of 22 immune cell contents in the control and PAH samples from the training set. **C** Correlations between the 4 biomarkers and 22 immune cell types. **D** Correlation analysis between the expression level of DDIT3 and abundance of neutrophils (left), and the expression level of OSM and abundance of resting memory CD4 T cells (right) in the training set

**Fig. 7:**
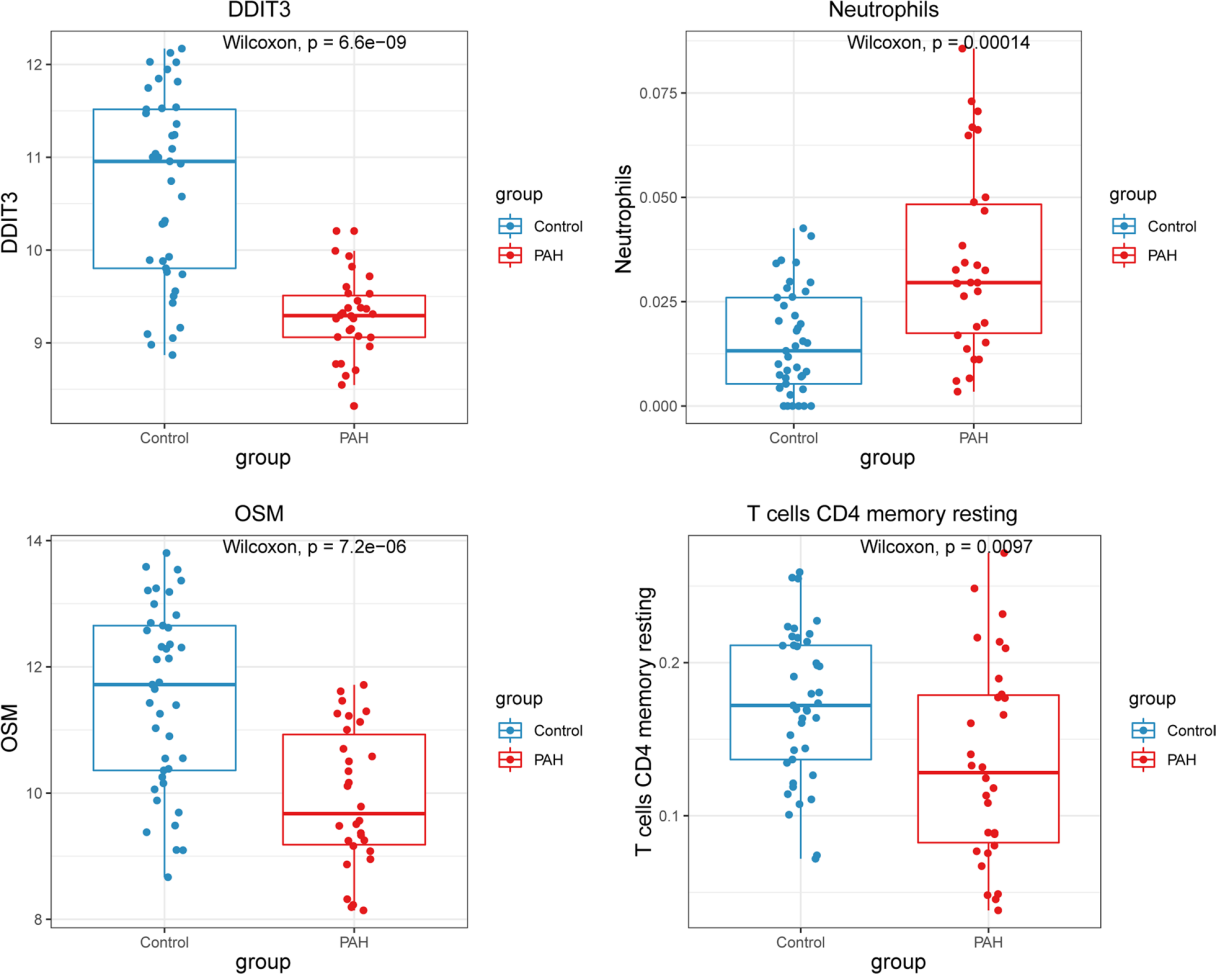
Wilcoxon’s test. Wilcoxon’s test of the expression of OSM and DDIT3, and the abundance of memory-resting CD4 T cells and neutrophils between the PAH and control samples

As immunity/inflammation is considered a critical pathogenesis mechanism during PAH development [20, 21]), we investigated the different immune/ inflammatory cells, and employed the CIBERSORT algorithm. Eight immune cell types were significantly different (*p* < 0.05) between the two groups, including naive B cells, memory B cells, resting memory CD4 T cells, follicular helpe rT cells, monocytes, M0 macrophages, M2 macrophages, and neutrophils (Fig. 6A–B). Among these immune cells, OSM had the most significant positive correlation with resting memory CD4 T cells, and DDIT3 had the most significant negative correlation with neutropils (Fig. 6C–D). Moreover, the expressions levels of OSM and DDIT3, and the abundance of resting memory CD4 T cells and neutropils were significantly different between the PAH and control samples (Fig. 7).

### Use of biomarkers for potential drug prediction using the DGIdb database

**Fig. 8:**
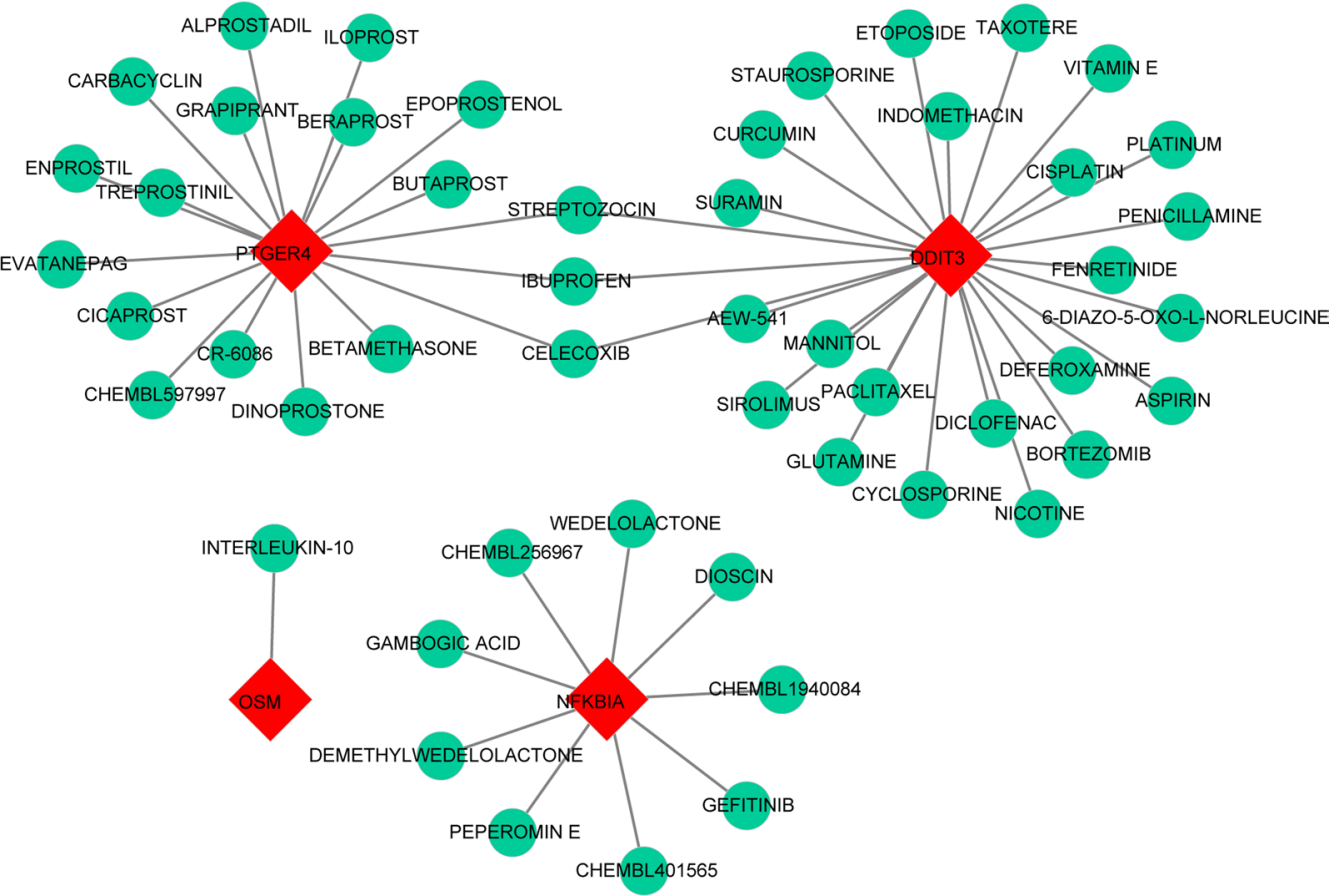
Drug–gene interaction diagram. The red square indicates the four biomarkers, and the green circle indicates the drugs

A gene-drug network was constructed and is displayed in Fig. 8. It was found that 27 drugs were predicted by DDIT3, 9 drugs were predicted by NFKBIA, 1 drug was predicted by OSM, and 21 drugs were predicted by PTGER4. Moreover, the network also suggested that STREPTOZOCIN, IBUPROFEN, and CELECOXIB were shared by PTGER4 and DDIT3.

### Verification of biomarker expression in clinical samples

**Fig. 9:**
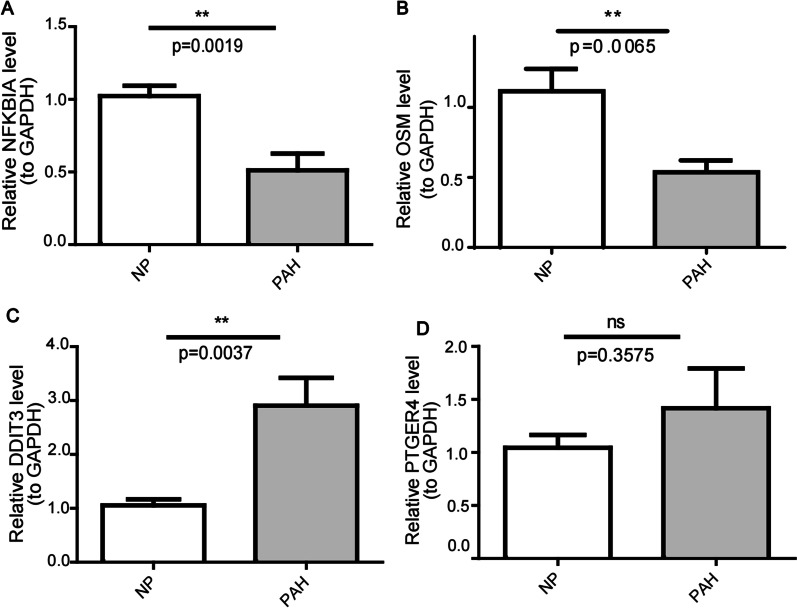
The expression of biomarkers (NFKBIA (**A**), OSM (**B**), DDIT3 (**C**), and PTGER4 (**D**)) in clinical PBMC samples detected by RT-qPCR. NP indicates normal peopole. **p value < 0.01

As illustrated in Fig. 3A, expression of DDIT3, NFKBIA, OSM, and PTGER4 was reduced in the PAH samples compared with control samples. We then further confirmed the expression in clinical samples (eight control samples and eight PAH samples) by RT-qPCR. In agreement with the results of the public database data analysis, NFKBIA and OSM were markedly down-regulated in clinical PAH samples versus control samples (Fig. 9). However, the trends in DDIT3 and PTGER4 expression were not consistent with the public database results, probably due to sample heterogeneity or limited sample size (Fig. 9).

## Discussion

As early as the 1980s, researchers found that increased serum copper may be a cause or a marker of PAH [22], and intravenous infusion of copper sulfate significantly increased pulmonary vascular resistance. [23], indicating an important role of copper during PAH development. It is critical for organisms to maintain homeostatic concentrations of copper. In recent years, copper metabolism has emerged as an important metabolic research direction for PAH. However, the mechanism underlying the effects of copper and changes in copper metabolism in PAH remain to be studied. To overcome these problems, this study screened differentially expressed copper metabolism-related genes. Four-gene-based models were constructed, and DDIT3, NFKBIA, OSM, and PTGER4 had improved diagnostic value in identifying PAH compared with normal controls, and thus had potential to be biomarkers for PAH. The immune infiltration profiles of PAH and normal controls were significantly different. High proportions of memory B cells, monocytes, M0 macrophages, M2 macrophages, and neutrophils were found in PAH, while high proportions of resting memory CD4 T cells, naïve B cells, and follicular helper T cells were found in normal controls. OSM was most positively correlated with resting memory CD4 T cells, and DDIT3 was most negatively correlated with neutrophils; Drugs were predicted by targeting the 4 biomarkers, and STREPTOZOCIN, IBUPROFEN, and CELECOXIB were shared by PTGER4 and DDIT3.

The copper metabolism-related genes DDIT3, NFKBIA, OSM, and PTGER4 were downregulated in PAH, as identified by WGCNA and SVM. Copper could cause increased expression of DDIT3 [24], DDIT3, as an oncogene [25], has been reported to promote vascular remodeling in MCT-induced PH [26], but its expression and role in PAH have not been studied. In this study, DDIT3 was upregulated in the validated cohort of PAH patients, which is not consistant with our predicted results and requires further studies to understand the possible pathogenic mechanisms as a controversial point. NFKBIA, OSM, and PTGER4, which were predicted to be downregulated in PAH by bioinformatics analysis in the present study have not yet been reported in PAH. The prediction results of NFKBIA and OSM are consistent with the validated results, while the expression of PTGER4 was not significantly changed in the validated cohort of PAH patients. Our study indeed provides new research directions for PAH. Usually, NFKBIA functions as a tumour suppressor and has the potential to be introduced as a novel anti-tumour agent [27, 28], whether it can reverse pulmonary vascular remodeling in PAH is not yet clear. It is known that nuclear factor-κB (NF-κB) plays an important role in PAH [29], which could be regulated by copper [30, 31]. Inhibition of NF-κB, prevents MCT-PH in mice [32]. NFKBIA serves as an inhibitor [33, 34] in regulating NF-κB and is reported to suppress the epithelial-mesenchymal transition (EMT), cell migration, proliferation and invasion [27]. Our results indicated an important role of NFKBIA in PAH development, which provides a new research direction for PAH that needs to be clarified further. OSM, a member of the interleukin 6 cytokine family, can suppress fibroblast activation to prevent cardiac fibrosis by inhibiting the SMAD signaling pathway [35]. OSM treatment preserved cardiac function and inhibited apoptosis and fibrosis after myocardial infarction [36]. In addition, OSM protected against cardiac I/R injury by regulating apoptosis, insulin sensitivity and mitochondrial biogenesis in diabetic mice [37]. Some studies have reported that OSM activates endothelial cells [38] and smooth muscle cells [39], but to date, the role of OSM in PAH and the underlying mechanisms remain to be studied. There is an imbalance between vasodilation and vasoconstriction favouring vasoconstriction with an increase in circulating vasoconstrictors and a decrease in circulating vasodilators (i.e., prostacyclin and prostaglandin) during PAH development. Therefore, prostacyclin and prostaglandin analogs are crucial treatments for PAH [40]. The prostaglandin receptor PTGER4 agonist could attenuate PAH by activating PPARγ [41] and suppressing EndMT [42]. The PAH pharmacotherapies beraprost and iloprost can bind to PTGER4 to mediate vasodilatory functions [43, 44]. A previous study showed that although the prostacyclin receptor was downregulated, PTGER4 had a stable expression [44], which is consistent with our validated results. Studies have also shown increased PTGER4 expression in pulmonary artery aneurysm with dissection in a patient with PAH [45]. However, in this study, we predicted that PTGER4 was downregulated in PAH. Thus, the expression pattern of PTGER4 in different subgroups of PAH need to be researched further.

GSEA enrichment analyses demonstrated key pathways involved in PAH, indicating that the citrate cycle (TCA cycle) participates in PAH development. This finding is consistent with previous studies, and abnormal TCA cycle flux occurrs in PAH [46]. Additional studies should focus on metabolic dysregulation in PAH to offer powerful therapeutic means to prevent or even reverse disease progression at the molecular level.

Immune cells play an indispensable role in the process of pulmonary hypertension vessel remodeling [47]. Therefore, attention should be given to the mechanism of immune cell infiltration in patients with PAH. We found that memory B cells, monocytes, M0 macrophages, M2 macrophages and neutrophils were increased in PAH, while resting CD4 memory T cells, naïve B cells and follicular helper T cells were decreased. A previous study reported that B lymphocytes are involved in vessel biology, vasomotor regulation, angiogenesis and cell proliferation [48]. Macrophages can cause vasoconstriction, increase vascular permeability, and induce proliferation [20]. Different subsets of CD4 + T cells play different roles in PAH, including small pulmonary artery muscularization, initiation and maintenance of inflammation, promotion of vascular remodeling, suppression of vascular inflammation, and limitation of the propagation of vascular injury [49]. Our previous study demonstrated the crucial role of interleukin 17–producing CD4^+^ effector T cells in hypoxia-induced PH [50], while CD4^+^ regulatory T cells showed a protective role against PAH [51]. Myeloid cells, specifically nonclassical monocyte lineage cells promote vascular remodeling [52]. It is not yet fully elucidated how these immune cells alterations are involved in PAH, and the molecular mechanisms involved in their activation remain unknown. Our results showed that OSM was most positively correlated with resting memory CD4 T cells, and DDIT3 was most negatively correlated with neutropils, laying the foundation for studying the immune mechanisms of PAH. The possible interaction mechanisms need to be clarified further.

Current PAH treatments generally target vasoconstriction by three different modalities: nitric oxide → soluble guanylate cyclase → cGMP levels, and the endothelin and prostacyclin pathways [53]. The treatment strategy for PAH has thereby changed significantly over the past decade, combination therapy has progressively become the gold standard of care in patients with PAH and is becoming widely used in clinical practice [54]. In this study, we identified 4 hub copper metabolism-related genes and constructed a gene-drug network to obtain agonists and antagonists of these molecules. The results provide a reference for clinicians to decide which drugs should be used in combination with current targeted drugs to improve patient prognosis and which drugs should be used with caution to prevent the clinical deterioration of PAH. The gene-drug network in this study illustrated that STREPTOZOCIN, IBUPROFEN, and CELECOXIB were shared by PTGER4 and DDIT3. Among them, IBUPROFEN and CELECOXIB are non-steroidal anti-inflammatory drugs (NSAIDs) that are commonly used in the clinic, and it has been reported that NSAIDs consumption during pregnancy contributes to an increased risk of persistent pulmonary hypertension of the newborns [55, 56]. Physicians should be alert to the potential dangers of these drugs to PAH patients.

This study uncovers a link between copper metabolism-related genes and pulmonary hypertension, highlighting several potential biomarkers. These biomarkers have the potential to be used as a routine diagnostic strategy and in the evaluation of PAH patients. Copper metabolism has potential as a new diagnostic biomarker as well as a targeted therapy for PAH, while the underlying mechanisms need to be clarified further in future studies.

## Conclusions

In summary, this study identified four copper metabolism-related biomarkers (DDIT3, NFKBIA, OSM, and PTGER4) with considerable diagnostic values based on bioinformatics analyses, and further constructed a gene-drug network. The results of this study may have significant implications for the development of new diagnostic biomarkers and actionable targets to expand treatment options for PAH patients.

## Supplementary Information

**Additional file 1. Supplementary Table 1.** Basic characteristics of the CTD-PH patients and the healthy subjects.

**Additional file 2. Supplementary Table 2.** The ROC values and 95% confidence intervals of 28 module DE-CMGs.

## Data Availability

The data analysed during this study are available in the GEO database (https://www.ncbi.nlm.nih.gov/geo/; GSE33463 and GSE113439) and the GeneCards database (https://www.genecards.org/).

## Abbreviations

PAH: Pulmonary arterial hypertension
GEO: The Gene Expression Omnibus
CMGs: Copper metabolism-related genes
DE-CMGs: Differentially expressed copper metabolism-related genes
DEGs: Differentially expressed genes
ROC: Receiver operating characteristic
AUC: The area under the curve
SVM: Support vector machine
LASSO: The least absolute shrinkage and selection operator
GSEA: Gene set enrichment Analysis
DGIdb: Drug-Gene Interaction Database
RT-qPCR: Real-time quantitative PCR
PH: Pulmonary hypertension
WGCNA: Weighted gene co-expression network analysis
CIBERSORT: Cell type identification by estimating relative subsets of RNA transcripts
PBMC: Peripheral blood mononuclear cell
GO: Gene Ontology
BP: Biological process
CC: Cellular components
MF: Molecular functions
KEGG: Kyoto Encyclopedia of Genes and Genomes
FC: Fold change
NF-κB: Nuclear factor-κB
EMT: Epithelial-mesenchymal transition
