# Supplementary material for: Identification of biomarkers related to copper metabolism in patients with pulmonary arterial hypertension
Source: BMC Pulm Med. 2023 Jan 23;23:31. doi: 10.1186/s12890-023-02326-6 (PMC9868507; doi:10.1186/s12890-023-02326-6)
Supplement: Supplementary file 1 — Additional file 1. Supplementary Table 1. Basic characteristics of the CTD-PH patients and the healthy subjects. [file 12890_2023_2326_MOESM1_ESM.docx]

Supplementary Table 1-1 Basic characteristics of the CTD-PH patients

| Subject | Age (Year) | Sex | sPAP (mmHg) | Protopathy |
| --- | --- | --- | --- | --- |
| 1 | 37 | Female | 65 | Systemic Lupus Erythematosus |
| 2 | 20 | Female | 62 | Systemic Scleroderma |
| 3 | 46 | Female | 43 | MCTD |
| 4 | 51 | Female | 47 | Systemic Lupus Erythematosus |
| 5 | 38 | Female | 118 | Takayasu Arteritis |
| 6 | 42 | Female | 41 | Systemic Lupus Erythematosus |
| 7 | 49 | Female | 44 | Systemic Lupus Erythematosus |
| 8 | 35 | Female | 97 | Systemic Lupus Erythematosus |

Supplementary Table 1-2 Basic characteristics of the healthy subjects

| Subject | Age (Year) | Sex |
| --- | --- | --- |
| 1 | 36 | Female |
| 2 | 42 | Female |
| 3 | 60 | Female |
| 4 | 22 | Female |
| 5 | 55 | Female |
| 6 | 22 | Male |
| 7 | 37 | Female |
| 8 | 47 | Female |
